# Articles You Might Have Missed

**DOI:** 10.1007/s13181-024-01011-2

**Published:** 2024-06-04

**Authors:** Taylor Sanders, D. Lynne McKee, E. Mason Jackson

**Affiliations:** 1 Atrium Health’s Carolinas Medical Center, Department of Emergency Medicine, Division of Medical Toxicology, Medical Education Bldg., 3rd floor, 1000 Blythe Boulevard, Charlotte, NC 28203 USA; 2 Prisma Health Upstate, Department of Emergency Medicine, 701 Grove Rd., 4th Floor Support Tower, Greenville, SC 29605 USA

**Keywords:** Noninvasive Positive Pressure Ventilation, Lead Exposure, Angiotensin II, Acetaminophen-induced Nephrotoxicity

## Article #1

Freund et al. Effect of Noninvasive Airway Management of Comatose Patients with Acute Poisoning: A Randomized Clinical Trial. JAMA. 2023;330(23):2267-2274.

### Background

Decreased level of consciousness in patients can potentially lead to respiratory insufficiency, hypoxemia and aspiration of gastric contents leading to worsening clinical outcomes and potentially death. The Glasgow Coma Score (GCS) provides an objective measurement of a patient’s mental status assessing the patient’s eyes, vocal response, and motor function. This score is frequently used in the setting of trauma and a score of eight of less is concerning for airway compromised necessitating intubation. In contrast, patients suffering from an acute poisoning event often have a depressed mental status that leads to intubation, a less than benign intervention. Despite the prevalence of poisoning induced intubations, no high level of evidence exists to determine which poisoned patents with decreased mental status require intubation.

### Research Question

What are the effects of withholding initial intubation among suspected poisoned patients with a GCS <9?

### Methods

This was a unblinded, randomized, parallel-group multicenter trial conducted at 20 Emergency Departments and one Intensive Care Unit (ICU) involving suspected acute poisoning (i.e., overdose) patients with a GCS of <9. Subjects were randomized into two groups: control = intubation was left to the discretion of the treating physician; intervention = intubation as withheld for four hours of close observation and tracking of vital signs (e.g., blood pressure, oxygen saturation and GCS). Exclusion criteria included the following: suspicion that the poisoning involved a cardioactive (e.g., beta blocker) or reversible (i.e., opioid) drug, emergent intubation criteria (including seizure, pulse oximetry <90% on nasal cannula, vomiting, or shock), age < 18 years, pregnancy, or incarceration. Primary outcomes included in-hospital mortality, ICU length of stay, and total hospital length of stay; secondary outcomes included mechanical ventilation at 28 days or discharge, number of intubated days, ICU admission rate, rapid-onset pneumonia, and complications from intubation. Subgroup analyses were performed on subjects with a GCS <7 and those with suspected GABA-agonist poisoning. Statistical analyses used the Finklestein-Schoenfeld method (win-loss-tie), Mann-Whitney U tests and linear regression models; statistical significance was set at a two-sided p value < 0.05.

### Results

A total of 225 subjects were analyzed; 109 controls and 116 in the intervention group. Intubation occurred in 57.8% of controls versus 16.4% of the intervention (restricted intubation) group; more controls were admitted to the ICU compared to the intervention group (66.1% v 39.7%). The most common (often only suspected) agents involved (controls %; intervention %, respectively) included alcohol (65.1%; 68.1%), benzodiazepines (40.4%; 38.8%), neuroleptics (28.4%; 19%), and GHB/GBL (10.1%; 12.1%). Compared to routine (control) management, restrictive intubation resulted in lower median ICU stay (0 h [IQR 0-18.5] v 24 h [IQR 16-79]) and median hospital stay (21.5 h [IQR 10.5-44.5] v 37 h [IQR 16-79]). Patients in the restrictive intubation group experienced fewer intubation-associated complications and a 7.8% reduction in rapid onset pneumonia. There were no deaths.

### Conclusion

Among the patients enrolled in this study, delaying immediate intubation of suspected poisoned patients with decreased levels of conscious was safe and associated with lower rates of ICU admission and complications.

### Critique

Confirmation of poisoning (qualitatively and or quantitatively) was not required. The lack of deaths suggests selection (less sick) bias. The majority of cases involved (suspected) GABA-agonists (including alcohol or GHB) poisoning, which often results in transient clinical effects and might explain the apparent benefit of conservative (restrictive intubation) initial management.

### Implication for Toxicologists

Delaying immediate intubation of acute overdose patients with decreased level of consciousness may be appropriate in some patients. More prospective work, involving confirmed overdoses, is required to establish such guidelines.

## Article #2

Mayer M, Feng T, Sukut, S, et al. Blood and hand surface lead in veterinary workers using lead shielding during diagnostic radiography. J Occup Environ Med. 2023;65(9):794-797.

### Background

Veterinary workers may experience lead exposure due to the wearing of lead shielding during radiologic procedures that, compared to human providers, usually involved use of lead-containing gloves and prolonged use during restraint of the animal during imaging. Damage to the lead shieling is also common in the veterinarian setting. While absorption of lead through skin contact is negligible, there is a risk of dermal-oral contact once lead shielding articles are removed.

### Research Questions

Are blood lead levels (BLL) higher in veterinary workers who regularly handle lead shielding compared to veterinary workers who do not? Does the use of disposable gloves under lead gloves reduce dermal lead exposure?

### Methods

Non-pregnant workers from a veterinary college were recruited and categorized as “exposed” if they reported any handling of lead shielding in the previous three months, those reporting no handling of lead shield (for three months) were used as controls. A questionnaire was used to assess occupational behaviors (e.g., use of lead and/or disposable gloves) and identify non-occupational lead exposures. Blood was collected and analyzed using a reference range of ≤ 0.48 umol/L for women and 0.72 umol/L for men; the level of detection was 0.01 umol/L (Note: 0.48 umol/L ~ 10 ug/dL).

Hand surface lead levels were tested, using convenience sampling, via wipes of workers’ hands immediately before and after wearing lead gloves for radiographs while noting the use (or absence) of disposable gloves under the lead gloves. For control purposes, five sample collecting wipes were removed from the packaging and immediately placed in collection tubes.

### Results

A total of 61 subjects were enrolled, some of whom participated in both the BLL and hand wipe study. Of the 55 completing the exposure survey, 16 (26% of all subjects) reported non-occupational lead expose (e.g., home remodeling, firing range, soldering). A total of 53 workers were evaluated for BLL, 28 exposed and 25 controls. The median BLL for both groups was 0.03 umol/L; the ranges being 0.01-0.09 umol/L for controls and 0.01-0.39 umol/L for exposed.

For the hand wipe experiment, 51 samples were collected from 21 workers before and after the use of lead gloves and showed a significantly lower lead amount present before (median 2.71 ug; range 0.27-41.5) compared to after (median 13.77 ug; 0.28-2337.09) glove use (*p*<0.001). Those not wearing disposable gloves (*n*=26) had significantly more lead on their hands compared to those wearing disposable gloves (median 869.65 ug; range 8.8-2337.09 v median 5.47 ug; range 0.28-44.31, respectively [*p*<0.001]).

Eighteen participants reported handling lead shielding earlier in the day and before donning gloves; their median lead (wipe) level was significantly higher than those not handling lead shielding prior to glove use: 6.08 ug (range 1.53-24.36) v. 1.36 ug (range 0.27-41.4); *p*<0.001.

### Conclusions

Use of lead shielding during veterinarian work is associated with lead exposure. The use of disposable gloves while working with/using lead shielding can significantly reduce this lead exposure.

### Critique

Many important variables were addressed via questionnaire, introducing potential bias. Although acknowledged by the authors, dermal lead exposure (without dermal-to-oral transmission) is of limited clinical importance. Related to this, the effects of hand washing were not evaluated. Not all participants answered the survey or engaged in both the BLL and hand wipe aspects of the study.

### Implication for Toxicologists

Understanding occupational lead (and other) exposures must include evaluation of workplace duties and practices, and well as individuals’ potential non-occupational exposure. Use of lead shielding during medical imaging is a source of potential lead exposure, use of appropriate personal protective equipment (i.e., disposable gloves) and hand washing should be encouraged.

## Article #3

Smith L, Mentz G, Engoren MC. Angiotensin II for the treatment of refractory shock: a matched analysis. Crit Care Med. 2023;51(12):1674-1684.

### Background

Angiotensin II (AT2) was approved for the treatment of distributive shock in 2017 after the Angiotensin II for the Treatment of Vasodilatory Shock (ATHOS-3) trial demonstrated significant improvement in blood pressures of patients receiving AT2 for catecholamine-refractory shock vs. placebo. While this study did not find a difference in mortality, and the invasive methods employed are impractical in most institutions, AT2 is increasingly used for rescue therapy for refractory shock in fashions inconsistent with the original study. Since publication, subgroup analyses of the ATHOS-3 data have shown mortality reductions in select subgroups, but the study overall was underpowered for mortality conclusions to be made. A retrospective study of 147 patients also showed no mortality reduction with the addition of AT2 as a third line vasopressor, but it too was underpowered. With the appeal of AT2 for distributive shock accelerating its use, given its unique mechanism and receptors, resolution of its association with mortality should be determined via an adequately powered study.

### Research Question

Does the addition of AT2 improve 30-day mortality in patients with severe, refractory shock requiring high doses of vasopressors?

### Methods

This was a retrospective cohort study of patients admitted with shock to the ICU of a university-based health system between 2016 and 2022. The primary outcome measure was all-cause 30-day mortality, comparing patients receiving any combination of norepinephrine, epinephrine, phenylephrine, vasopressin, or dopamine to those patients additionally receiving AT2. It was noted that AT2 was typically used only for shock refractory to the other listed vasopressors. All adult patients receiving vasopressors were initially included, however patients receiving AT2 initiated in the operating room were excluded. Patients receiving AT2 were then matched to controls based on norepinephrine equivalent (NE) doses at the time AT2 was initiated. The control group was further divided into historical and concurrent controls, defined by vasopressors being administered before or after AT2 became available on March 14, 2018. Baseline characteristics were obtained within 24 h of enrollment and mean arterial pressure (MAP) and Sequential Organ Failure Assessment (SOFA) scores were calculated for five days post-enrollment. Etiology of shock was determined by preset criteria as either septic, hemorrhagic, vasoplegic without sepsis, or other. Secondary outcomes included 90-day mortality and post-enrollment initiation of renal replacement therapy (RRT) and mechanical ventilation. Tertiary outcomes included mortality-adjusted SOFA scores (MA-SOFA), ventilator-free days, and major thrombotic events within 30 days (defined as venous thromboembolism, acute coronary syndrome, mesenteric ischemia, or stroke). Standard exploratory data analysis, univariate comparison with standardized differences (SDiff) and chi-square and independent *t* tests, and multivariate analysis were performed. The Generalized Estimating Equation method was used to test the hypothesis, that AT2 exposure did not affect 30-day mortality, to account for correlation amongst repeated observations. Results from ATHOS-3 were used to determine a total of 594 patients, 198 AT2 patients with two controls for each, were necessary to achieve 90% power for determining a similar difference in mortality.

### Results

A total of 271 patients receiving AT2 were included in the study group, with 542 matched (concurrent and historical) controls. The mean patient age was 61 (+/- 16) years and most were male (60%) and white (81%). Pre-enrollment NE equivalent dosing was comparable between AT2 and controls, with SDiff (effect size) of 0.004. The median duration of AT2 treatment was 0.7 (interquartile range, 0.2-1.9) days. The 30-day mortality was similar between the AT2 and control groups (60% vs. 56%; SDiff = 0.079; *p* = 0.292). The 90-day mortality was also similar for AT2 and controls (65% vs. 63%; SDiff = 0.058; *p* = 0.440). Baseline higher SOFA scores, lower MAPs, and a diagnosis of septic shock were all significantly more prominent in the AT2 group. This group also had significantly higher creatinine levels, higher rates of chronic illness, and were more likely to have already received mechanical ventilation or RRT at enrollment. After accounting for these baseline characteristics, no association was found between AT2 and the primary outcome or any of the secondary and tertiary outcomes. Thirty-day mortality was associated with several factors including older age, higher baseline NE equivalent doses, higher lactate, and RRT prior to enrollment. Higher MAP at enrollment and postoperative state were associated with decreased 30- and 90-day mortality.

### Conclusion

AT2 was not associated with improved mortality or organ dysfunction when used as salvage therapy for patients with severe shock, nor was it associated with an increase in adverse events.

### Critique

Unlike ATHOS-3 and other prior studies, this retrospective cohort study was sufficiently powered to detect a mortality difference. However, a large proportion of patients were diagnosed with septic shock. While logistic regression did not show an association between AT2 and mortality in septic patients, the AT2 patients were significantly older with more diagnostic results and interventions typically associated with worsened disease states. Most of these baseline characteristics were associated with higher 30- and 90-day mortality on multivariate analysis, creating the possibility of residual confounding despite the analytical methods. The study methods did not control for AT2 or other vasopressor dosing and the mean maximum AT2 dose (35.2 ng/kg/min) was less than half the approved maximum initial dose (80 ng/kg/min). Altogether, potential variability in practice could lead to underestimation of benefit or adverse effects from AT2. The NE equivalent dosing during and after the observation period were not reported, leaving to question any benefit from AT2 regarding dose reduction of other vasopressors. Finally, the study only examined AT2 as salvage therapy for refractory shock; further studies are needed to determine the role for AT2 as a second- or first-line agent.

### Implication for Toxicologists

AT2 should be considered as adjunctive treatment for refractory shock once first-line agents (e.g., norepinephrine) have been appropriately titrated for effect. Of note, similar to other vasopressors, the vasoconstrictive effects of AT2 are dependent upon functioning L-type calcium channels in vascular smooth muscle cells.

## Article #4

Mazer M, Perrone J. Acetaminophen-induced nephrotoxicity: pathophysiology, clinical manifestations, and management. J Med Toxicol. 2008;4(1):2-6.

### Background

Acetaminophen-induced nephrotoxicity (APAP-N) has been observed in humans (and animal studies) in both acute and chronic supratherapeutic ingestions, manifesting in 1-2% of patients. The pathophysiology and clinical course of the resultant renal insufficiency may differ from APAP-induced hepatotoxicity; the causative and protective effects of N-acetylcysteine (NAC) on APAP-N are unknown.

### Research Questions

What is known about the pathophysiology and clinical features of renal insufficiency in the setting of acute APAP toxicity? How should APAP-N be managed?

### Methods

This narrative review discussed several aspects of acute renal insufficiency in the setting of APAP toxicity, including possible pharmacologic and pathophysiologic mechanisms, histopathology, reported clinical manifestations and course, relationship to other variables (e.g., dose and degree of hepatotoxicity), and management. A single case report was used to present data and recommendations along with references to human and animal studies, case reports, and retrospective poison center reviews.

### Results

The single case report involved a 47-year-old woman with a history of fibromyalgia and gastric bypass but no history of alcohol use disorder or renal insufficiency, who was found by family with altered mentation and recent reported ingestion of nine grams of APAP over two days. She presented with lethargy, tachycardia (HR 112) and tachypnea (RR 32); significant laboratory results included an APAP concentration of 12 mcg/ml, AST 5,409 u/L, ALT 1,085 u/L, creatinine of 0.9 mg/dL, and a urine drug screen positive for marijuana and opiates; she had no hematuria, proteinuria or ketonuria. Oral NAC was started but she developed fulminant hepatic failure with coagulopathy (INR 5.1), hypoglycemia, and an AST of 11,840 u/L on hospital day (HD) three. She was intubated for persistent obtundation, and creatinine increased to 2.3 mg/dL with BUN of 10 mg/dL; repeat APAP concentration was 2 mcg/mL. Creatinine continued to rise and peaked at 8.1 ng/dL on HD12. She ultimately required multiple days of hemodialysis (HD) but was extubated and ultimately discharged on HD27, at which time her creatinine had improved to 1.3 mg/dL.

Animal studies have demonstrated that NAPQI is formed via CYP 2E1 in nephrocytes during excess APAP exposure, exerting similar toxic effects to that of the liver once glutathione is depleted. Other potential mechanisms of APAP-N include NAPQI formation via prostaglandin endoperoxidase synthetase, seen in both animal and human studies, as well as animal studies revealing the formation of p-aminophenol via N-deacetylase, a free radical precursor. Regardless of mechanism, renal biopsies in humans have shown that the proximal tubule is the most prominent site of toxic effects, resulting in a histopathologic pattern consistent with acute tubular necrosis (ATN). Human case reports have shown that this ATN-like renal injury, similar to hepatic injury, is more likely to occur in those at risk for CYP 2E1 upregulation or glutathione depletion. Interestingly, and with unclear reason, adolescents and young adults may also be more at risk. In these case reports, most cases of APAP-N began between two and five days post-exposure, with creatinine levels peaking at an average of seven days and returning to baseline in most cases within a month. Retrospective studies from poison centers reported almost a third of patients who developed renal insufficiency had no evidence, at the time, of liver injury. Case reports and retrospective studies have not shown a difference in peak creatinine levels among patients receiving NAC for APAP-N, and animal studies have failed to show a protective effect from NAC.

### Conclusion

APAP-N occasionally occurs after supratherapeutic APAP ingestion and can occur in the absence of hepatotoxicity. It typically follows a pattern similar to ATN and, while most patients make a full recovery, temporary HD may be necessary. NAC has not been shown to prevent or improve nephrotoxicity; further research is needed.

### Critique

Utilizing a case report for illustration, this review article presents a thorough review of the clinical manifestations and pathophysiology of APAP-N. The review includes clinically relevant data and discussion of the correlation between APAP-N and dose, as well as the overall lack of evidence supporting NAC for prevention or treatment in the absence of hepatotoxicity. While there are limitations of summary reviews, the conclusions from this article continue to prevail since its publication 16 years ago, as demonstrated in the 2023 Consensus statement by ACMT, AACT, America’s Poison Centers, and the Canadian Association of Poison Centers.

### Implication for Toxicologists

This article remains a comprehensive resource on APAP-N for both insight and clinical guidance. Recent literature has suggested a possible protective role for 4-methylpyrazole in APAP-N but further research is needed. The breadth of data continues to support the futility of NAC and other interventions for the treatment or prevention of APAP-N.

